# Effects of Sodium Bicarbonate Ringer’s Solution Combined with Positive End-Expiratory Pressure on the Internal Environment of Patients Undergoing Bariatric Surgery: A Randomized 2 × 2 Factorial Design Pilot

**DOI:** 10.1007/s11695-024-07631-5

**Published:** 2024-12-26

**Authors:** Xinyue Jiang, Rui Wang, Lan Guo, Zhengru Shan, Zhiping Wang

**Affiliations:** 1 Xuzhou Medical University, Xuzhou, China; 2 Department of Anesthesiology, The Affiliated Hospital of Xuzhou Medical University, Xuzhou, China

**Keywords:** Sodium bicarbonate Ringer’s solution, Positive end-expiratory pressure, Acid–base balance, Postoperative recovery quality

## Abstract

**Background:**

To evaluate the influence of sodium bicarbonate Ringer’s solution (BRS) combined with positive end-expiratory pressure (PEEP) on the internal environment in patients who have undergone laparoscopic bariatric surgery.

**Methods:**

A total of 128 patients undergoing laparoscopic bariatric surgery were randomly divided into the control group (group C), the PEEP group (group P), the BRS group (group B), and the BRS combined with the PEEP group (group BP). The results of arterial blood gas analysis, including pH value, base excess (BE), concentrations of electrolyte, and lactate (Lac) were documented before intravenous infusion (T0) and 5 min after the surgery (T1). Additional metrics included tumor necrosis factor-α (TNF-α) and interleukin-6 (IL-6) and were quantified before intravenous infusion and at 30 min post-surgery. The quality of recovery-15 questionnaire (QoR-15) scores were documented preoperatively (D0) and on the first (D1) and third (D3) days, postoperatively.

**Results:**

There was no significant interaction effect between the two factors of BRS and PEEP (*p* = 0.659). After the infusion of BRS, the pH level increased significantly at T2 (*p* < 0.05). Using PEEP during operation can increase PaO2 in patients with obesity (*p* < 0.05). The level of pH value is increased, and the concentrations of inflammatory factors are reduced due to the combination of BRS and PEEP (*p* < 0.05). Compared with group C, group BP exhibited an augmentation in QoR-15 (*p* < 0.05), and the recovery time of group BP was significantly shortened (*p* < 0.05).

**Conclusions:**

BRS combined with PEEP has been demonstrated to improve acid–base balance, reduce the inflammatory response, shorten the recovery time, and substantially enhance the quality of early postoperative recovery.

## Background

Obesity is a common disease, and with the development of the economy and changes in lifestyle and dietary habit, its incidence is increasing rapidly [1]. Patients with obesity contain a large amount of adipose tissue, and 50 ~ 70% of the intake of glucose will be converted into Lac in adipose tissue; moreover, the increase of adipose tissue is always accompanied by an increase in Lac production [2].On the other side, patients undergoing gastrointestinal surgery are prone to fluid loss and decreased circulating blood volume due to long-term abstinence from drinking and fasting, mechanical intestinal preparation, and vasodilator effect of general anesthetics [3, 4]. Hence, these patients are more likely than a healthy person to have insufficient tissue oxygen supply and increased glycolysis and Lac concentration and generate metabolic acidosis. Because of tissue hypoperfusion and Lac accumulation, patients with obesity are also at increased risk of complications such as hypotension, inflammation, acid–base imbalance, and organ dysfunction [5]. Consequently, for patients with obesity, further research is urgently needed to identify and develop effective interventions to improve their internal environment and accelerate rapid recovery after surgery.

BRS is a new type of electrolyte solution. Compared with sodium lactate Ringer’s solution and acetic acid Ringer’s solution, it has more advantages in the replenishment of extracellular fluid in the decrease of interstitial fluid and circulating blood flow, the reduction of Lac concentration, and the correction of metabolic acidosis [6]. PEEP has also been shown to reduce the production of injurious inflammatory cytokines in the blood of patients with obesity and to maintain the acid–base balance of the internal environment [7]. However, few studies have reported that the combination of the two can improve the internal environment of patients with obesity. Therefore, the purpose of this study was to explore the effects of BRS combined with PEEP on acid–base balance in patients undergoing bariatric surgery, in order to provide reference for clinical practice.

## Methods

### Study Design

The previous studies have shown that the mean pH values after operation in groups P and B were 7.40 and 7.39, with standard deviations of 0.034 and 0.092, respectively [7, 8]. Based on clinical pretrial results, it is preliminarily estimated that the mean pH values of group BP and group C were 7.34 and 7.32, with standard deviations of 0.050 and 0.060. The significance level was set at 0.05, and the desired power of the test was established at 90%. Using PASS software version 15.0, the calculated sample size per group was determined to be 28 individuals, amounting to a total of 112 participants for the four groups combined. To account for a potential dropout rate of 10%, the total sample size was adjusted to 128 participants (*n* = 128). This adjustment ensures an adequate sample size to maintain the statistical power of the study despite possible participant attrition.

This investigation is a single-center, prospective, randomized, double-blind controlled, factorial design trial, which has been approved by the Ethics Committee of the Affiliated Hospital of Xuzhou Medical University (XYFY2024-KL013-01). A total of 128 patients undergoing elective bariatric surgery in the Department of Bariatrics and Metabolic Surgery, Affiliated Hospital of Xuzhou Medical University from May to August 2024, were selected. Inclusion criteria include patients undergoing bariatric surgery, BMI ≥ 30 kg/m^2^, aged 18–65 years old, on gender limitation, and ASA grades II–III. Exclusion criteria include patients with severe heart, brain, lung, liver, kidney, and metabolic dysfunction; PEEP contraindications (bullae of lung, emphysema, etc.); those with severe obstructive sleep apnea–hypopnea syndrome; those with a previous history of upper abdominal surgery and pulmonary surgery; and those who refuse to sign the informed consent. Rejecting criteria included intraoperative colloid infusion, the intraoperative blood loss was more than 2000 ml, the operation time was more than 3 h, a second tracheal intubation was required, patients were admitted to ICU after operation, and the operation was canceled for various reasons. According to the random number table method, they were divided into four groups (*n* = 32): the control group (group C), the PEEP group (group P), the BRS group (group B), and the BRS combined with PEEP group (group BP). The patient’s assigned number was placed in an opaque, airtight bag, which was opened by an acupuncturist who was not involved in this surgery or data collection, and grouped accordingly to the grouping inside the envelope. The members of the surgery, data collectors, statisticians, and patients involved in this study were unaware of the grouping.

### Procedures

Preoperative protocols entailed an 8-h fast and drink and intestinal preparation. Upon entering the operating room, routine monitoring of electrocardiography, non-invasive arterial pressure, peripheral capillary oxygen saturation, mean arterial pressure (MAP), and heart rate (HR) was initiated. Invasive blood pressure monitoring was facilitated through radical artery catheterization under local anesthesia, utilizing heparin for anticoagulation and enabling blood sample collection. After that, peripheral venous access was established. During anesthesia induction, head height and foot height were adopted, and 100% pure oxygen was inhaled through the mask. Patients received routine rapid sequence intravenous anesthesia induction using midazolam (0.05 mg/kg), sufentanil (0.5 µg/kg), and etomidate (0.3 mg/kg). Rocuronium (0.6 mg/kg) is given to facilitate tracheal intubation. After intubation, transversus abdominis plane block was performed under ultrasound guidance. Combined intravenous and inhaled anesthesia was maintained with propofol, remifentanil, sevoflurane, and vecuronium until the end of surgery. Post-intubation, mechanical ventilation was initiated under direct laryngoscopic guidance, employing a pressure control-volume guaranteed ventilation mode with a tidal volume set between 6 and 8 ml/kg and an inspiratory expiratory ratio of 1:1. The oxygen flow rate was maintained at 2 l/min with an inspired oxygen concentration of 60%, and the respiratory rate was adjusted to sustain the arterial blood carbon dioxide partial pressure within the range of 35–45 cmH2O. For patients in group P and BP, they received an additional 10 cmH2O PEEP.

Only compound sodium chloride injection (Nacl) was injected in groups C and P and only BRS was injected in groups B and BP. Preoperative fluid loss was calculated according to the 4–2-1 rule, half of the fluid was added before induction, half was added before the establishment of pneumoperitoneum, and then the crystal fluid was continuously transfused 6–8 ml/kg/h (according to the ideal weight calculation: male, [height (cm) − 100] × 0.9; female, [height (cm) − 100] × 0.85). A MAP decrease of more than 20% from baseline was addressed by administering 5 mg of ephedrine or 40–80 µg of phenylephrine intravenously. If hypotension recurs, 250–500 ml colloidal fluid is rapidly administered until MAP recovers. Sevoflurane was discontinued 20 min before the end of the procedure. At the conclusion of surgery, the use of propofol and remifentanil was stopped, while antagonists were administered intravenously, and the tracheal tube was removed after indications of extubation appeared. The patients were then provided with oxygen via a post-anesthesia care unit (PACU) mask and transferred back to the ward once they reached the Aldrete score of more than 9 points. Post-pain was administered intravenously by a clinician not involved in the study, according to the patient’s needs.

### Outcome Measures

Baseline data was meticulously gathered for the study, including arterial blood gas analysis results. The primary outcome was a pH value 5 min after surgery. Secondary outcomes included the other results of arterial blood gas analysis, such as BE and PaO2, and the concentration of ions like Na + , K + , Cl − , Ca2 + , HCO3-, Lac, and glucose (Glu) concentrations. Arterial blood samples were collected at 30 min post-operation for the evaluation of plasma levels of inflammation. These included TNF-α and IL-6, both of which were measured using ELISA. Additionally, we also recorded the QoR-15 scores on D0, D1, and D3. Post-extubation assessments at 24 h included the visual analogue scale (VAS) for pain and analgesic drug use, as well as the score for postoperative nausea and vomiting (PONV). Recovery index such as recovery time after the operation was also recorded. All observational indicators were recorded by physicians who were blinded to the group assignments of the patients to ensure the integrity and unbiased nature of the data collected.

### Statistical Analysis

Data analysis was conducted using SPSS version 25.0 software. The normality of continuous variables was examined using the Shapiro–Wilk test. Normally distributed continuous variables were expressed as mean ± standard deviation (SD). Factorial design data were analyzed using a two-way analysis of variance. For between-group comparisons, one-way analysis of variance (ANOVA) was employed, while repeated measures ANOVA was utilized for within-group comparisons. Continuous variables that do not satisfy the normal distribution are represented by the median (M) and quartile intervals (P25.P75), and the measurement data in multiple groups were compared with the Kruskal–Wallis *H* test. Categorical variables were analyzed using the *χ*^2^ test or Fisher’s exact test as appropriate. Post hoc analysis was performed using Bonferroni correction when multiple groups were statistically significant. A *p*-value of less than 0.05 was considered indicative of statistical significance.

## Result

**Fig. 1:**
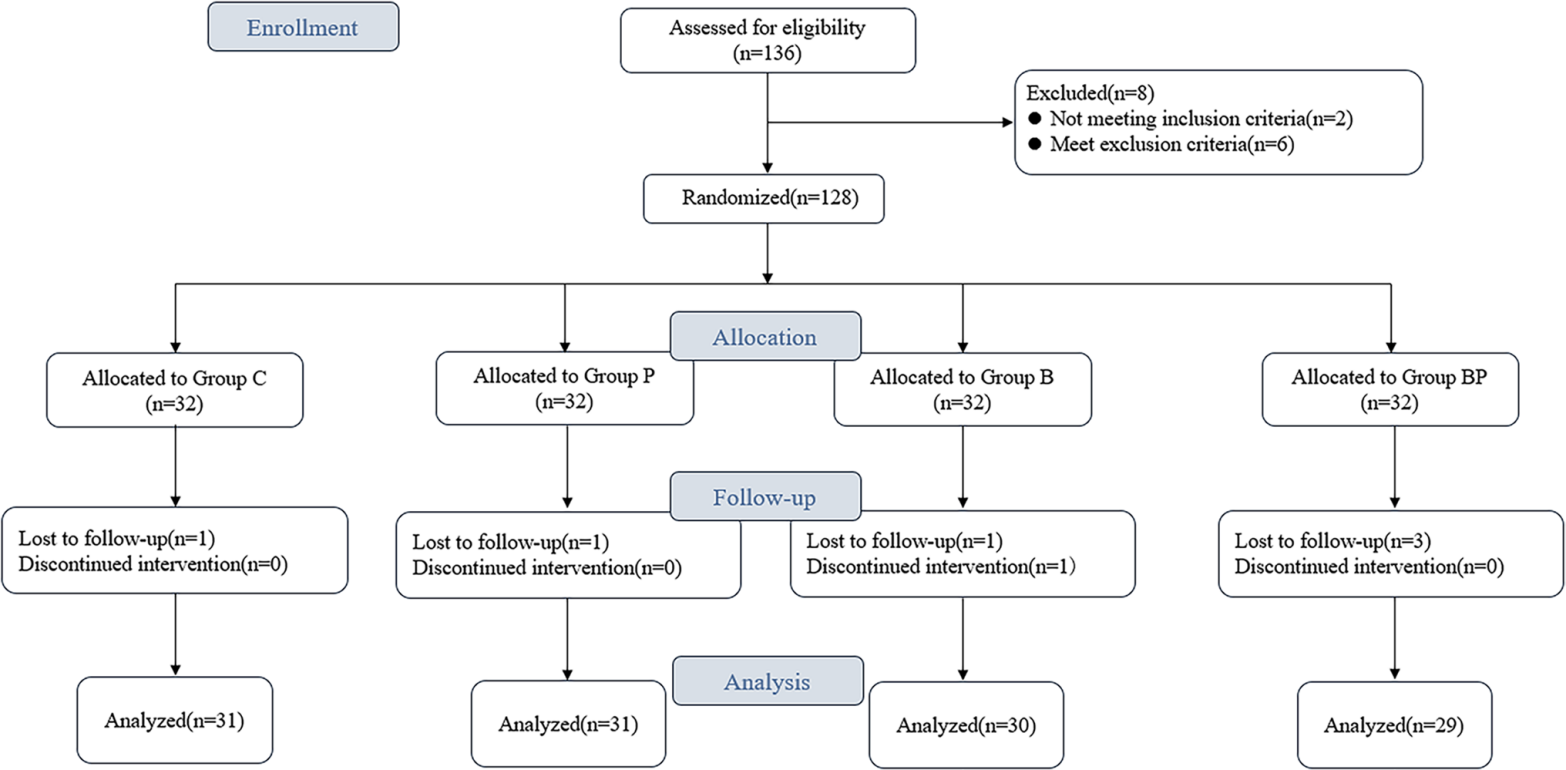
CONSORT diagram

In the current investigation, an initial cohort of 128 patients was considered for inclusion. However, 7 patients were subsequently excluded from the study due to various reasons: 4 cases were removed due to the operation that lasted more than 3 h; 1 patient needed to be reintubated after surgery; intervention was discontinued in 1 patient due to excessive intraoperative bleeding; and 1 patient was unable to cooperate with the postoperative evaluation protocols. Consequently, the statistical analysis was conducted on a final sample of 121 patients, with the distribution as follows: 31 in group C, 31 in group P, 30 in group B, and 29 in group BP. The flow of participant inclusion and exclusion is illustrated in Fig. 1. There was no significant difference in general data among the four groups (*p* > 0.05) (Table 1).

**Table 1:** Baseline characteristics of study participants

	Group C (*n* = 31)	Group P (*n* = 31)	Group B (*n* = 30)	Group BP (*n* = 29)		F/*x*^2^/H	*p* value
Age (years, *x̄* ± s)	33.32 ± 8.03	33.52 ± 9.33	32.67 ± 8.84	28.48 ± 7.31		2.334	0.078
Patient sex, male (*n*, %)	16 (51.6)	15 (48.4)	9 (30.0)	9 (31.0)		4.840	0.184
ASA classification (*n*, %)						2.900	0.407
II	13 (41.9)	11 (35.5)	17 (56.7)	13 (44.8)			
III	18 (58.1)	20 (64.5)	13 (43.3)	16 (55.2)			
Height (cm, *x̄* ± s)	171.97 ± 8.28	171.58 ± 7.65	168.23 ± 8.21	169.21 ± 10.02		1.362	0.258
Weight (kg, *x̄* ± s)	121.08 ± 23.47	119.10 ± 20.97	111.33 ± 18.64	114.27 ± 24.82		1.233	0.301
BMI (kg/m^2^, *x̄* ± s)	40.77 ± 6.60	40.28 ± 5.43	38.73 ± 5.34	39.39 ± 5.67		0.751	0.524
Diseases of the patients at admission							
Hepertension (*n*, %)	13（41.9）	10 (32.3)	6 (20.0)	9（31.0）		3.420	0.331
Diabetes mellitus (*n*, %)	4 (12.9)	8 (25.8)	6（20.0）	8 (27.6)		2.376	0.498
Surgery time [min, M (P25, P75)]	129 (97,151)	112 (90,137)	113.5 (100,138)	105 (81,136)		6.056	0.109
Intraoperative propofol used (mg, *x̄* ± s)	375.32 ± 140.24	333.19 ± 101.33	326.83 ± 99.88	320.00 ± 92.54		1.146	0.337
Intraoperative remifentanil dosage (mg, *x̄* ± s)	3.39 ± 0.97	3.18 ± 1.05	2.95 ± 0.84	3.07 ± 0.84		1.205	0.311
Cumulative fluid volume (ml, *x̄* ± s)	1059.29 ± 239.51	1056.00 ± 227.52	1056.60 ± 253.57	1164.07 ± 165.33		1.662	0.179
Preoperative index							
MAP (mmHg, *x̄* ± s)	104.45 ± 13.63	103.00 ± 12.59	101.47 ± 9.18	103.86 ± 13.96		0.327	0.806
HR (times/min, *x̄* ± s)	77.81 ± 5.13	79.52 ± 7.77	78.97 ± 6.00	77.93 ± 6.83		0.491	0.689
QoR-15 score (scores, *x̄* ± s)	138.26 ± 6.24	140.52 ± 7.63	141.03 ± 6.29	141.00 ± 4.29		1.346	0.263
Arterial blood gases							
pH (*x̄* ± s)	7.41 ± 0.02	7.41 ± 0.03	7.42 ± 0.02	7.42 ± 0.02	2.304	0.080	
PaO2 (mmHg, *x̄* ± s)	91.44 ± 29.88	97.47 ± 38.50	88.84 ± 8.51	98.44 ± 33.57	0.724	0.540	
Na + (mmol/l, *x̄* ± s)	140.32 ± 1.99	139.70 ± 2.26	139.90 ± 2.09	139.83 ± 1.81	0.532	0.662	
Cl- (mmol/l, *x̄* ± s)	105.65 ± 2.37	109.53 ± 16.91	106.60 ± 2.88	109.86 ± 18.98	0.823	0.484	
K + (mmol/l, *x̄* ± s)	3.76 ± 0.30	3.69 ± 0.29	3.73 ± 0.27	3.59 ± 0.25	1.996	0.118	
Ca2 + (mmol/l, *x̄* ± s)	1.13 ± 0.06	1.13 ± 0.06	1.14 ± 0.05	1.13 ± 0.04	0.213	0.887	
HCO3- (mmol/l, *x̄* ± s)	25.70 ± 2.75	24.42 ± 1.97	24.72 ± 2.14	24.52 ± 1.89	2.153	0.097	
BE (mmol/l, *x̄* ± s)	0.51 ± 1.45	−0.69 ± 2.16	0.37 ± 2.06	−0.11 ± 2.21	2.282	0.083	
Lac (mmol/l, *x̄* ± s)	1.16 ± 0.52	1.08 ± 0.43	1.01 ± 0.49	0.99 ± 0.52	0.725	0.539	
Glu (mmol/l, *x̄* ± s)	5.60 ± 0.81	5.75 ± 1.51	5.46 ± 0.73	5.67 ± 1.43	0.423	0.737	

*ASA* American Society of Anesthesiologists, *BMI* body mass index, *pH* potential of hydrogen, *BE* base excess, *Lac* lactate, *Glu* glucose

**Fig. 2:**
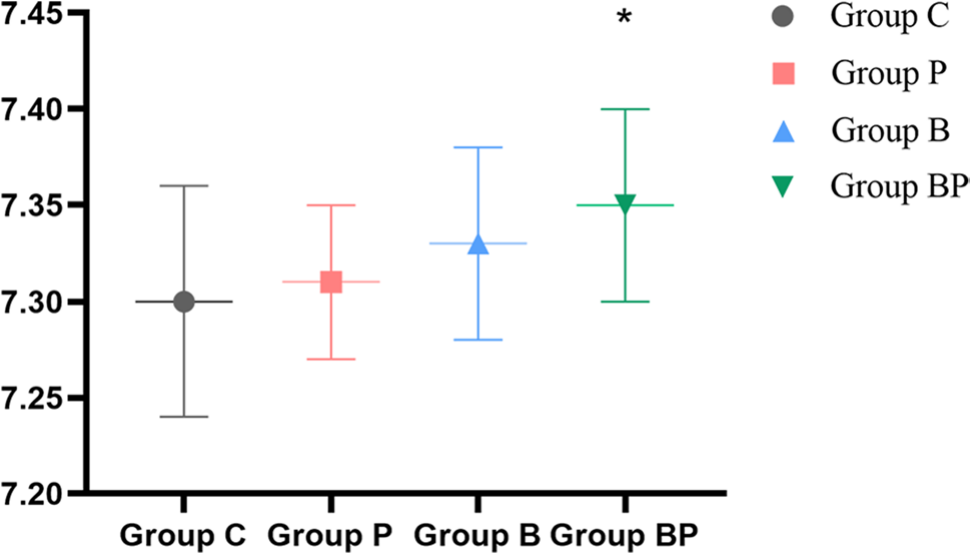
Comparison of pH value 5 min after operation; *p < 0.05 vs. group C

When analyzing the interaction of factors, no interaction effect was found among the factors (*p* = 0.659), indicating that the effects of BRS and PEEP were independent of each other, and the main effects of BRS and PEEP should be analyzed. Compared with the use of Nacl, BRS significantly increased the pH value of patients undergoing laparoscopic bariatric surgery (*p* < 0.05); there was no statistically significant improvement in pH value after surgery with PEEP compared with zero PEEP (ZEEP) (*p* > 0.05) (Table 2). Subsequently, we used one-way ANOVA for postoperative pH value and found that BRS combined with PEEP exhibited an augment in postoperative pH value compared to Group C (*p* < 0.05) (Fig. 2).

**Table 2:** Comparison of pH value 5 min after operation

Index	Group BRS (*n* = 59)	Group NaCl (*n* = 62)	*p* value	Group PEEP (*n* = 60)	Group ZEEP (*n* = 61)	*p* value
pH value	7.34 ± 0.01*	7.31 ± 0.01	0.001	7.33 ± 0.01	7.32 ± 0.01	0.109

Using two-way analysis of variance; data are presented as *x̄* ± s

*p < 0.05 vs. group NaCl

**Fig. 3:**
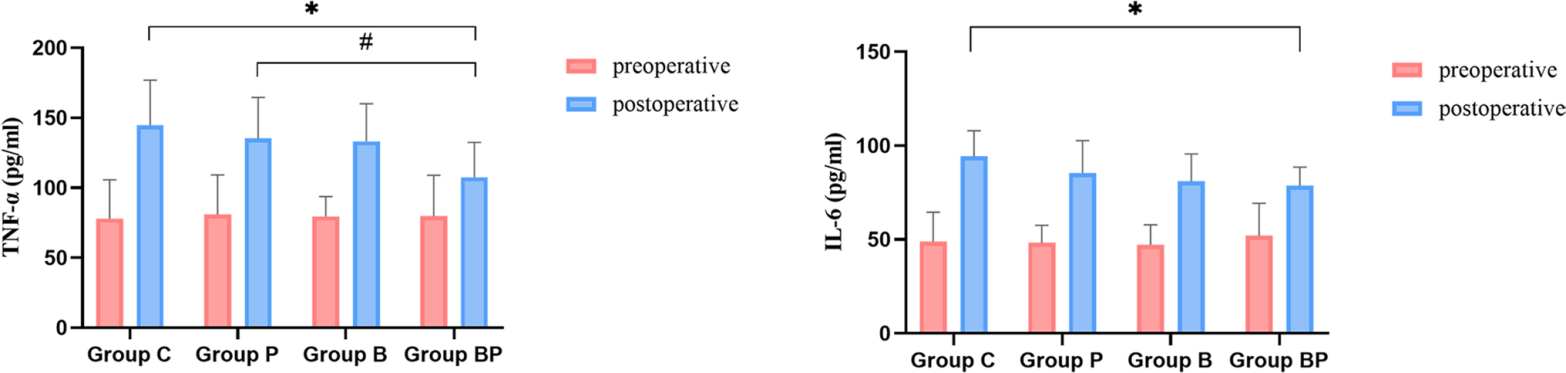
Comparison of serological markers among the four groups; *p < 0.5 vs. group C; #*p* < 0.05 vs. group P

The two factors also had no interaction effect with other blood gas analysis results and inflammatory factors. The main effect of BRS showed a decrease in concentrations of Na + , K + , Ca2 + , Lac, TNF-α, and IL-6 (*p* < 0.05) and an increase in concentration of HCO3- and BE. Compared with ZEEP, PaO2 was increased and the concentration of HCO3- and TNF-α decreased after operation with PEEP (*p* < 0.05) (Table 3). When both BRS and PEEP were used, the levels of TNF-α and IL-6 were decreased postoperatively compared with group C (*p* < 0.05) (Fig. 3).

**Table 3:** Comparison of other arterial blood gas analysis indicators and inflammatory factors

Index	Group BRS (*n* = 59)	Group NaCl (*n* = 62)	*p* value	Group PEEP (*n* = 60)	Group ZEEP (*n* = 61)	*p* value
PaO2	244.80 ± 15.01	231.00 ± 14.64	0.512	265.42 ± 14.77	210.38 ± 14.89^#^	0.010
Na +	140.03 ± 0.19*	140.79 ± 0.19	0.006	140.15 ± 0.19	140.67 ± 0.19	0.059
Cl −	106.09 ± 1.19	109.21 ± 1.16	0.062	108.61 ± 1.18	106.69 ± 1.17	0.247
K +	3.87 ± 0.06*	4.05 ± 0.06	0.024	3.90 ± 0.06	4.03 ± 0.06	0.091
Ca2 +	1.15 ± 0.01**	1.18 ± 0.01	< 0.001	1.16 ± 0.01	1.17 ± 0.01	0.461
HCO3 −	24.37 ± 0.30*	23.46 ± 0.30	0.033	23.48 ± 0.30	24.34 ± 0.30^#^	0.046
BE	− 1.74 ± 0.27*	− 2.95 ± 0.26	0.002	− 2.61 ± 0.27	− 2.08 ± 0.27	0.160
Lac	0.80 ± 0.05*	0.94 ± 0.05	0.042	0.85 ± 0.05	0.89 ± 0.05	0.542
Glu	5.95 ± 0.16	5.88 ± 0.16	0.737	5.94 ± 0.16	5.89 ± 0.16	0.820
TNF-α	120.39 ± 4.88*	139.98 ± 5.03	0.007	121.43 ± 4.95	138.95 ± 4.95^#^	0.015
IL-6	79.84 ± 2.44*	89.85 ± 2.55	0.006	82.05 ± 2.48	87.65 ± 2.51	0.118

Using two-way analysis of variance; data are presented as *x̄* ± s, *TNF-α* tumor necrosis factor-α, *IL-6* interleukin-6

*p < 0.05 vs. group NaCl, **p < 0.001 vs. group NaCl, ^#^*p* < 0.05 vs. group ZEEP

**Fig. 4:**
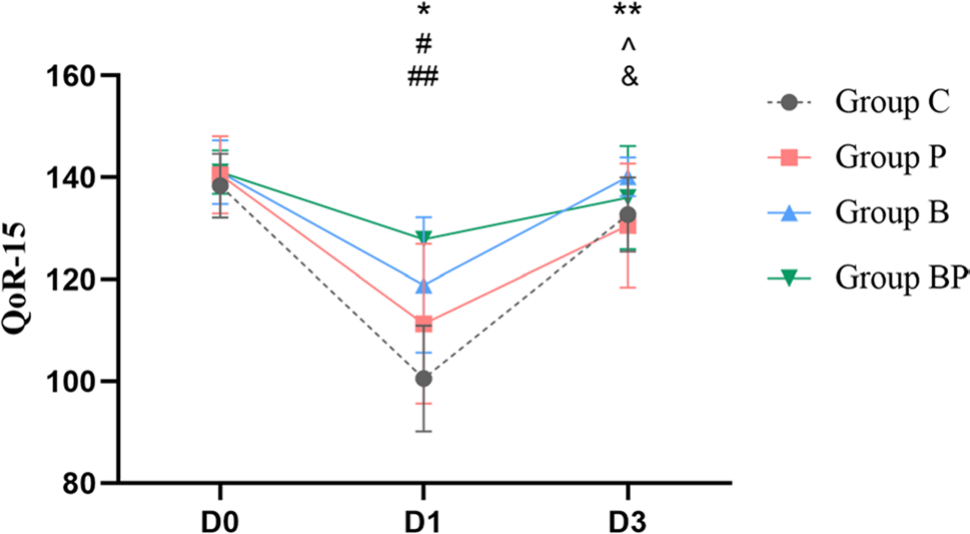
Comparison of QoR-15 scores in four groups; *p < 0.05, four groups vs. D0; **p < 0.05, groups C and D vs. D0; ^*p* < 0.05, groups C, P, and B vs. D1; #*p* < 0.05 vs. group C; ##*p* < 0.05, groups P and B vs. group BP; &*p* < 0.05, groups C and P vs. group B

There were statistically significant differences in QoR-15 scores (*p* < 0.05). Compared with D0, four groups exhibited a reduction in QoR-15 scores on D1 (*p* < 0.05); and both group C and group P showed decreases in QoR-15 scores on D3 (*p* < 0.05). Group C, group P, and group B experienced an increase in QoR-15 scores on D3 in comparison with D1 (*p* < 0.05). When comparing with group C at the same points, three groups exhibited an increase in QoR-15 scores, and group BP had the highest score on D1 (*p* < 0.05). Comparing with group B at the same points, group C and group P had a decrease in QoR-15 scores on D3 (*p* < 0.05) (Fig. 4).

Across the four groups, no statistically significant differences were noted in several postoperative conditions: duration of stay in PACU, the postoperative length of stay, the VAS pain scores 24-h post-operation, the consumption of dezocine on the first-day post-operation, and the PONV scores (*p* > 0.05). It is remarkable, however, that group B and group BP reported shorter awakening times compared to group C (*p* < 0.05), and group B and group BP also showed shorter awakening times than group P (*p* < 0.05) (Table 4).

**Table 4:** Postoperative conditions of participants

	Group C (*n* = 31)	Group P (*n* = 31)	Group B (*n* = 30)	Group BP (*n* = 29)	*F*/*H*	*p* value
Awakening time (min, *x̄* ± s)	12.74 ± 3.18	11.71 ± 3.59	8.67 ± 3.01*^#^	8.00 ± 3.24*^#^	15.006	< 0.001
PACU residence time (min, *x̄* ± s)	39.00 ± 12.93	32.16 ± 9.45	33.40 ± 14.87	31.83 ± 9.51	2.327	0.083
Postoperative length of stay [days, *M* (P25, P75)]	2 (2,3)		2 (2,2)		4.246	0.236
VAS pain scores at 24 h after operation (score, *x̄* ± s)	5.68 ± 2.29	5.18 ± 2.48	4.83 ± 1.93	5.32 ± 2.82	0.656	0.581
PONV score at 24 h after operation (score, *x̄* ± s)	2.94 ± 1.75	2.86 ± 1.78	3.00 ± 1.66	2.32 ± 1.65	0.866	0.461
Dezocine dose during the first postoperative day [mg, M (P25, P75)]	0 (0,10)	0 (0,5)		0 (0,0)	6.259	0.100

^*^p < 0.05 vs. group C

^#^*p* < 0.05 vs. group P

**Fig. 5:**
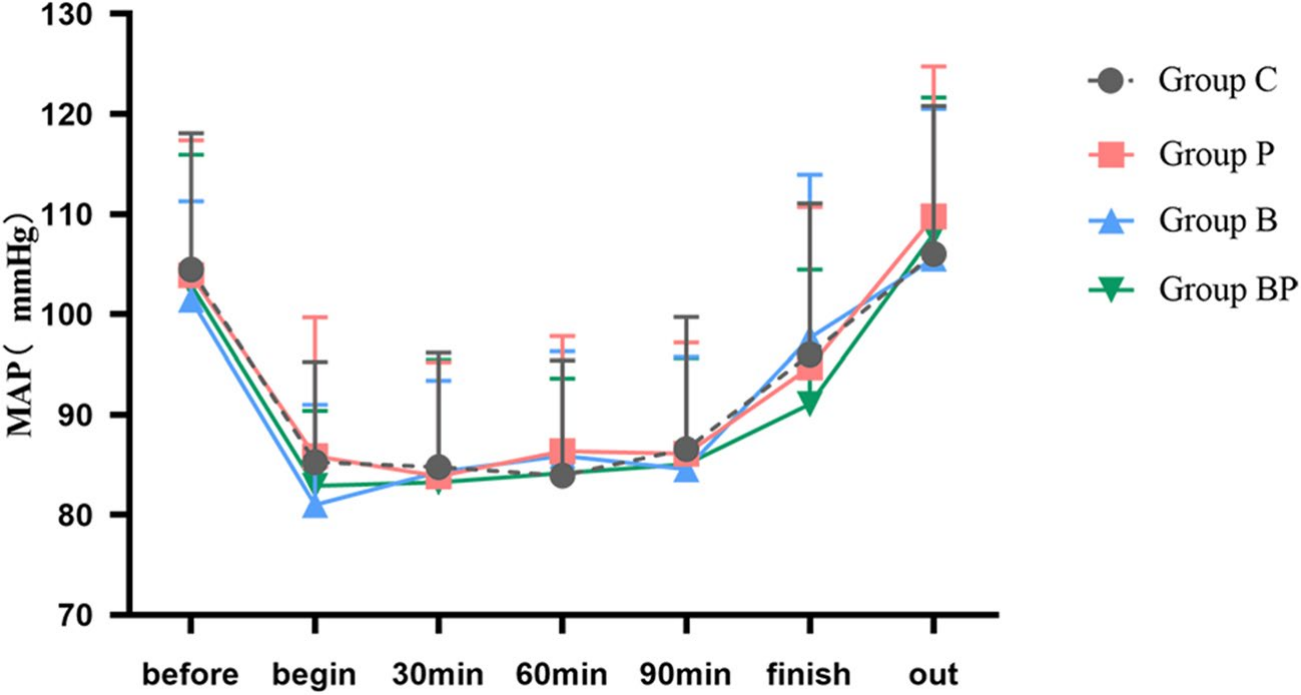
Comparison of mean arterial pressure in 4 groups of participants

Hemodynamic outcome, such as MAP, was not statistically different among the four groups (Fig. 5).

## Discussion

The aim of this study was to investigate the effect and impact of BRS combined with PEEP on acid–base balance and inflammatory response in patients undergoing laparoscopic bariatric surgery. Surprisingly, we observed a significant beneficial effect of BRS combined with PEEP on improving acid–base balance. On the other hand, serological experiments also revealed significant changes in TNF-α and IL-6 postoperatively in patients with BRS and PEEP. In addition, the combination of BRS and PEEP can shorten the time to recovery and significantly improve the quality of early recovery after surgery, and there is no interaction effect between BRS and PEEP.

Water, inorganic salt ions, acid–base equilibrium substances, inflammatory substances, and metabolites are all internal environmental components, which are essential for cell survival and the maintenance of normal physiological functions of cells. Previous studies have shown that obesity leads to impaired mitochondrial function in skeletal muscle, which means increased glycolysis and Lac production in muscle tissue [9, 10]; most of the Glu consumed by the body is converted to Lac in adipose tissue [2]. Therefore, patients with obesity are more likely to develop hyperlactatemia and metabolic acidosis than the able-bodied person. At the same time, adipocytes in the adipose tissue of patients with obesity can synthesize high levels of proinflammation cytokines, including TNF-α, LI-6, and IL-1β, which may induce systemic inflammation after entering the blood [11]. Consequently, it is very important to ensure the safe management of the perioperative period and promote the rapid recovery of patients.

Although the osmotic pressure produced by the commonly used crystal solution such as Nacl is similar to that of plasma, the concentration of sodium and chloride ions in the crystal solution is higher than that in plasma, and the excessive infusion can easily lead to hypernatremia and hyperchloremia [12, 13]. BRS, a new type of balanced crystalloid buffered with bicarbonate instead of organic anions which provides physiological levels of bicarbonate ions and electrolyte ions, can be used to supplement missing extracellular fluid and correct metabolic acidosis timely [14]. Not only that, but BRS can also reduce the body’s inflammatory response and maintain hemodynamic stability [6, 15]. In our study, the postoperative pH value of the BRS group was significantly higher than that of the NaCl group, and the absolute values of BE and Lac concentration were significantly lower than those of the NaCl group, indicating that compared with the infusion of NaCl, the intraoperative use of BRS can quickly and effectively replenish blood volume and reduce hypoxia and hypoperfusion caused by hypotension during anesthesia, and thus reduce the production of Lac in the body. Therefore, it can correct potential metabolic acidosis, which is also consistent with the findings of Wang et al. [16].

PEEP is one of the lung protective ventilation strategies and has been widely used in clinical practice. Since the glottis cannot be closed during mechanical ventilation, using PEEP during the surgery not only reduces end-expiratory alveolar collapse, thereby improving body oxygenation, but also reduces the incidence of ventilator-associated pneumonia and lung injury [17, 18]. The result of our trial showed that intraoperative use of PEEP at 10 cmH2O can improve postoperative PaO2 in patients undergoing laparoscopic bariatric surgery, reaffirming the beneficial effect of PEEP on postoperative oxygenation in patients with obesity. Although PEEP at a level of 10 cmH2O is the best level of PEEP for maintaining oxygenation in patients with obesity, excessive PEEP leads to hemodynamic instability [19]. The potential reasons for good hemodynamic stability lie in adequate fluid before maneuver [20]. During the surgery, no significant difference in MAP and HR in the four groups of patients was observed; thus, we speculated that the use of BRS may quickly replenish the missing extracellular fluid and maintain hemodynamic stability in time.

This study indicates that the pH value after operation can be improved by using BRS combined with PEEP. Perhaps it is because the bicarbonate in BRS can be directly alkalized and excreted by exhaling carbon dioxide (CO2), thereby regulating the pH value of the body to physiological levels [21]. Meanwhile, PEEP regulates postoperative pH value by improving body oxygenation and avoiding intraoperative and postoperative CO2 accumulation [7].

TNF-α, a sensitive index, reflects the severity of tissue injury [22]; IL-6 is an important index of early tissue damage [23]. The levels of TNF-α and IL-6 in arterial blood serve as valuable indicators for assessing the inflammatory response of the body [8]. The results of our study revealed that concentrations of TNF-α and IL-6 decreased significantly after resuscitation in BRS and usage of PEEP, suggesting that the combined use of the two measures could reduce inflammatory response in patients with obesity. Obesity is an independent risk factor for lung injury due to mechanical ventilation [24]. The use of PEEP can reduce the incidence of ventilator-associated lung injury. This may be because atelectasis can cause increased leakage of alveolar capillary protein and destruction of the vascular endothelium [25], and the use of PEEP reduces this effect. However, the molecular mechanism and signaling pathway of BRS to reduce the body’s inflammatory response are not clear, so further research is needed [15].

As a tool for measuring clinical intervention, the QoR-15 can briefly and effectively evaluate the impact of various factors on the quality of early postoperative recovery, and it is a reduced form of the Extended 40-item Questionnaire(QoR-40) [26]. The score ranges from 1 to 150, with higher scores indicating better recovery quality, and a difference of 6 points in QoR-15 is indicative of a meaningful change in health status [27]. This study’s findings highlight a statistically significant difference in QoR-15 scores among the four groups within the first 3 days post-surgery. Compared to group C, both infusions of BRS and usage of PEEP were observed to enhance QoR-15 scores on the first postoperative day, and the combination of them further amplified this improvement. Notably, there was no difference in QoR-15 scores on the third postoperative day in patients with BRS combined with PEEP. These results suggest that BRS and PEEP not only improve the quality of postoperative recovery, but also enhance the effect of combination of the two, and may restore the physical health status of the third to the preoperative level after the operation. Interestingly, previous studies have found that early postoperative hyperlacticemia, especially within 4 h, has adverse effects on the prognosis of patients, such as prolonged hospital stay and increased incidence of postoperative complications [28]. However, our study did not find any difference in the length of hospital stay and the incidence of postoperative complications between the four groups. The difference in the results of our analyses may be due to the age of patients before surgery, basic physiological status, and postoperative nursing guidelines, because the patients undergoing bariatric surgery are younger and have fewer underlying diseases. A stable internal environment is of great significance for early postoperative extubation [29]. The lactate level in arterial blood can reflect the function of various organs and the metabolic state of cells, which is very crucial for predicting the prognosis of patients [30]. This study also found that the recovery time of patients receiving BRS combined with PEEP was significantly shortened, which we thought might be because BRS improved the acidosis state of the body without increasing the concentration of exogenous lactic acid, alleviated the burden on the liver, enabled it to better play the detoxification function, and reduced the concentration of free propofol in blood. PEEP can avoid the accumulation of CO_2_ and thus shorten the wake time. This further indicates that BRS combined with PEEP can improve the postoperative recovery quality and accelerate the postoperative recovery of patients.

This study is a factorial design, which can observe the effects of multiple factors at the same time, improve the experimental efficiency, analyze the interaction between various factors, and find the best scheme or the best combination. Indeed, this study is not without its limitations. Firstly, the goal-directed fluid therapy was not adopted, which limited the rationality of intraoperative infusion volume; secondly, the collection of clinical data was limited to the first 3 days after surgery without long-term follow-up, which ignored the potential impact on patients’ long-term outcomes; finally, since fixed PEEP without considering the respiratory mechanics of individual patients is not the best choice [31], further studies on the efficacy of individualized PEEP combined with GDFT-guided BRS infusion in patients with obesity are needed.

## Conclusion

Our study confirmed that BRS combined with PEEP significantly improved acid–base balance, reduced the inflammation response, and shortened the recovery time, thereby substantially enhancing the quality of early postoperative recovery. These data suggested that the use of BRS combined with PEEP in laparoscopic bariatric surgery has positive results and is of high clinical value.

## Data Availability

No datasets were generated or analysed during the current study.

## References

[1] Hruby A, Hu FB. The epidemiology of obesity: a big picture. Pharmacoeconomics. 2015;33(7):673–89. 25471927 10.1007/s40273-014-0243-x PMC4859313

[2] Lin Y, Bai M, Wang S, Chen L, Li Z, Li C, et al. Lactate is a key mediator that links obesity to insulin resistance via modulating cytokine production from adipose tissue. Diabetes. 2022;71(4):637–52. 35044451 10.2337/db21-0535

[3] Pędziwiatr M, Mavrikis J, Witowski J, Adamos A, Major P, Nowakowski M, et al. Current status of enhanced recovery after surgery (ERAS) protocol in gastrointestinal surgery. Med Oncol (Northwood, London, England). 2018;35(6):95. 10.1007/s12032-018-1153-0 PMC5943369 29744679

[4] Bamboat ZM, Bordeianou L. Perioperative fluid management. Clin Colon Rectal Surg. 2009;22(1):28–33. 20119553 10.1055/s-0029-1202883 PMC2780230

[5] Liu J, Gao Y, He Z, Zhang H, Chen L. The efficacy of sodium bicarbonated Ringer’s solution versus lactated Ringer’s solution in elderly patients undergoing gastrointestinal surgery: a prospective randomized controlled trial. Am J Trans Res. 2023;15(8):5216–27. PMC10492062 37692958

[6] Hashemi SJ, Heidari SM, Yaraghi A, Seirafi R. Acid-base and hemodynamic status of patients with intraoperative hemorrhage using two solution types: crystalloid Ringer lactate and 1.3% sodium bicarbonate in half-normal saline solution. Adv Bbiomed Res. 2016;5:190. 10.4103/2277-9175.191000 PMC5156971 28028530

[7] Shi ZG, Geng WM, Gao GK, Wang C, Liu W. Application of alveolar recruitment strategy and positive end-expiratory pressure combined with autoflow in the one-lung ventilation during thoracic surgery in obese patients. J Thorac Dis. 2019;11(2):488–94. 30962992 10.21037/jtd.2019.01.41 PMC6409265

[8] Li Q, Yang Q, Tian C, Guo Y, Liu H, Cheng Y, et al. Effects of different types of Ringer’s solution on patients with traumatic haemorrhagic shock: a prospective cohort study. Eur J Med Res. 2024;29(1):215. 38566152 10.1186/s40001-024-01664-3 PMC10988935

[9] Berkemeyer S. Acid-base balance and weight gain: are there crucial links via protein and organic acids in understanding obesity? Med Hypotheses. 2009;73(3):347–56. 19410381 10.1016/j.mehy.2008.09.059

[10] Kristensen MD, Petersen SM, Møller KE, Lund MT, Hansen M, Hansen CN, et al. Obesity leads to impairments in the morphology and organization of human skeletal muscle lipid droplets and mitochondrial networks, which are resolved with gastric bypass surgery-induced improvements in insulin sensitivity. Acta Physiol (Oxf). 2018;224(4):e13100. 29791782 10.1111/apha.13100

[11] Zorena K, Jachimowicz-Duda O, Ślęzak D, Robakowska M, Mrugacz M. Adipokines and obesity. Potential link to metabolic disorders and chronic complications. Int J Mol Sci. 2020;21(10):3570. 32443588 10.3390/ijms21103570 PMC7278967

[12] Chiara O, Pelosi P, Brazzi L, Bottino N, Taccone P, Cimbanassi S, et al. Resuscitation from hemorrhagic shock: experimental model comparing normal saline, dextran, and hypertonic saline solutions. Crit Care Med. 2003;31(7):1915–22. 12847383 10.1097/01.CCM.0000074725.62991.42

[13] Wu CY, Chan KC, Cheng YJ, Yeh YC, Chien CT. Effects of different types of fluid resuscitation for hemorrhagic shock on splanchnic organ microcirculation and renal reactive oxygen species formation. Critical care (London, England). 2015;11(19):434. 10.1186/s13054-015-1135-y PMC4699328 26651994

[14] Li Q, Liu Y, Wang Y, Shan X, Liu C, Li Z, et al. Bicarbonate Ringer’s solution could improve the intraoperative acid-base equilibrium and reduce hepatocellular enzyme levels after deceased donor liver transplantation: a randomized controlled study. BMC Anesthesiol. 2023;23(1):418. 38114893 10.1186/s12871-023-02383-8 PMC10729548

[15] Han SJ, Zhou ZW, Yang C, Wei KP, Ma JZ, Chu ZF, et al. Hemorrhagic, hypovolemic shock resuscitated with Ringer’s solution using bicarbonate versus lactate: a CONSORT-randomized controlled study comparing patient outcomes and blood inflammatory factors. Medicine. 2022;101(46):e31671. 36401445 10.1097/MD.0000000000031671 PMC9678593

[16] Wang L, Lou J, Cao J, Wang T, Liu J, Mi W. Bicarbonate Ringer’s solution for early resuscitation in hemorrhagic shock rabbits. Annal Trans Med. 2021;9(6):462. 10.21037/atm-21-97 PMC8039674 33850859

[17] Hol L, Nijbroek S, Schultz MJ. Perioperative lung protection: clinical implications. Anesth Analg. 2020;131(6):1721–9. 33186160 10.1213/ANE.0000000000005187

[18] Lagier D, Zeng C, Fernandez-Bustamante A, Vidal Melo MF. Perioperative pulmonary atelectasis: part II. Clin Implications Anesthesiol. 2022;136(1):206–36. 10.1097/ALN.0000000000004009 PMC9885487 34710217

[19] Van Hecke D, Bidgoli JS, Van der Linden P. Does lung compliance optimization through PEEP manipulations reduce the incidence of postoperative hypoxemia in laparoscopic bariatric surgery? Randomized Trial Obes Surg. 2019;29(4):1268–75. 30612327 10.1007/s11695-018-03662-x

[20] Bohm SH, Thamm OC, von Sandersleben A, Bangert K, Langwieler TE, Tusman G, et al. Alveolar recruitment strategy and high positive end-expiratory pressure levels do not affect hemodynamics in morbidly obese intravascular volume-loaded patients. Anesth Analg. 2009;109(1):160–3. 19535706 10.1213/ane.0b013e3181a801a3

[21] Satoh K, Ohtawa M, Okamura E, Satoh T, Matsuura A. Pharmacological study of BRS, a new bicarbonated Ringer’s solution, in partially hepatectomized rabbits. Eur J Anaesthesiol. 2005;22(8):624–9. 16119600 10.1017/s0265021505001043

[22] Tan J, Song Z, Bian Q, Li P, Gu L. Effects of volume-controlled ventilation vs. pressure-controlled ventilation on respiratory function and inflammatory factors in patients undergoing video-assisted thoracoscopic radical resection of pulmonary carcinoma. J Tthoracic Dis. 2018;10(3):1483–9. 10.21037/jtd.2018.03.03 PMC5906281 29707298

[23] Schädler D, Pausch C, Heise D, Meier-Hellmann A, Brederlau J, Weiler N, et al. The effect of a novel extracorporeal cytokine hemoadsorption device on IL-6 elimination in septic patients: a randomized controlled trial. PLoS ONE. 2017;12(10):e0187015. 29084247 10.1371/journal.pone.0187015 PMC5662220

[24] Fernandez-Bustamante A, Hashimoto S, SerpaNeto A, Moine P, Vidal Melo MF, Repine JE. Perioperative lung protective ventilation in obese patients. BMC Anesthesiol. 2015;6(15):56. 10.1186/s12871-015-0032-x PMC4491899 25907273

[25] Wolthuis EK, Choi G, Dessing MC, Bresser P, Lutter R, Dzoljic M, et al. Mechanical ventilation with lower tidal volumes and positive end-expiratory pressure prevents pulmonary inflammation in patients without preexisting lung injury. Anesthesiology. 2008;108(1):46–54. 18156881 10.1097/01.anes.0000296068.80921.10

[26] Sethi N, Dutta A, Puri GD, Sood J, Choudhary PK, Gupta M, et al. Evaluation of quality of recovery with quality of recovery-15 score after closed-loop anesthesia delivery system-guided propofol versus desflurane general anesthesia in patients undergoing transabdominal robotic surgery: a randomized controlled study. Anesth Analg. 2024;138(5):1052–62. 38416594 10.1213/ANE.0000000000006849

[27] Campfort M, Cayla C, Lasocki S, Rineau E, Léger M. Early quality of recovery according to QoR-15 score is associated with one-month postoperative complications after elective surgery. J Clin Anesth. 2022;78:110638. 35033845 10.1016/j.jclinane.2021.110638

[28] Creagh-Brown BC, De Silva AP, Ferrando-Vivas P, Harrison DA. Relationship between peak lactate and patient outcome following high-risk gastrointestinal surgery: influence of the nature of their surgery: elective versus emergency. Crit Care Med. 2016;44(5):918–25. 26757164 10.1097/CCM.0000000000001567

[29] De Santis GC, Brunetta DM, Nardo M, Oliveira LC, Souza FF, Cagnolati D, et al. Preoperative variables associated with transfusion requirements in orthotopic liver transplantation. Trans Apheresis Sci Off J World Apheresis Assoc Off J Eur Soc Haemapheresis. 2014;50(1):99–105. 10.1016/j.transci.2013.10.006 24291115

[30] Yu LQ, Meng CC, Jin XS, Cai J. Clinical study of sodium bicarbonated Ringer’s solution on fluid resuscitation of patients with hemorrhagic shock. Eur Rev Med Pharmacol Sci. 2022;26(5):1535–42. 35302197 10.26355/eurrev_202203_28218

[31] Li X, Liu H, Wang J, Ni ZL, Liu ZX, Jiao JL, et al. Individualized positive end-expiratory pressure on postoperative atelectasis in patients with obesity: a randomized controlled clinical trial. Anesthesiology. 2023;139(3):262–73. 37440205 10.1097/ALN.0000000000004603

